# Availability of eye health interventions in basic schools in a Ghanaian municipality

**DOI:** 10.3389/fpubh.2024.1468285

**Published:** 2024-12-06

**Authors:** Christopher Senyo Adzaho, Emmanuel Appiah-Brempong, Princess Ruhama Acheampong, Ernest Ekutor

**Affiliations:** ^1^Department of Health Promotion and Disability Studies, School of Public Health, Kwame Nkrumah University of Science and Technology, Kumasi, Ghana; ^2^Eye Department, St John of God Hospital, Duayaw Nkwanta, Ghana

**Keywords:** school eye health, eye health intervention, child eye health, vision screening, eye health education, school health

## Abstract

**Introduction:**

Globally, 19 to 22 million children are visually impaired. A huge number of children therefore will not be able to learn effectively even if the best educational institutions are made available to them. This ultimately leaves a potential negative effect on their quality of life, educational opportunities and livelihoods. School health programs (SHPs) present a unique opportunity to provide comprehensive eye health services to children. This study assessed the availability of School Eye Health Programs (SEHPs) in a municipality in Ghana.

**Design and methods:**

In a cross-sectional study, semi-structured questionnaires and interview guides were used to collect data from 60 head teachers, 17 school health coordinators, and 7 key health workers.

**Results:**

No school in the municipality had a comprehensive SEHP being implemented. However, over 60% of schools had at some point introduced at least one component of SEHP, mainly Vitamin A supplementation and vision screening. Community and Public Health Nurses are the commonly used human resource for such programs. There were no available guidelines for program implementation and no systems in place for ensuring follow-up visits and provision of spectacles to those with refractive errors. Key barriers identified included the lack of financial resources and adequately trained personnel.

**Conclusion:**

Comprehensive school eye health programs are unavailable in the study area and there is an urgent need for their implementation to avert the potential adverse effects of vision impairment on the education of children.

## Introduction

Globally, more than 7 in every 100 children are visually impaired based on uncorrected visual acuity (1). Even though this number reduces drastically to 4 in every 100 children when presenting visual acuity is used, the situation remains one of great concern. This is because approximately 80% of human learning occurs through the sense of vision, which is closely associated with educational performance. Children who are visually impaired are more likely to have poor educational outcomes and are more likely to be out of school compared to their peers without visual impairment (2). The implication is that a significant number of children will struggle to learn effectively, even if the best educational institutions are available to them. The result is a potential negative effect on the quality of life, educational opportunities, and livelihoods of millions of children around the world (3, 4). In addition to poor educational performance, the 4% of children worldwide with visual impairment are at risk of developing permanent visual impairment if their conditions are not diagnosed early and managed appropriately (1). Early detection of eye conditions, especially refractive errors in children, is therefore key to enhancing their academic performance (5). The best and most cost-effective way of ensuring early detection of eye conditions and promoting good eye health among school-aged children is through school screening programs (6). The International Agency for the Prevention of Blindness (IAPB) has, in recent years, been focusing on and advocating for various child eye health interventions (7). For health interventions involving children, schools serve as an excellent and well-organized environment to access a large number of children. These interventions, commonly referred to as School Eye Health Programs (SEHP), include vision screening and refractive error correction (8), spectacle-wearing compliance (8), vitamin A supplementation, and eye health education for children, parents, and teachers (9). School health programs (SHPs) present an exceptional avenue to provide comprehensive eye health services, incorporating all components, to children in well-organized settings.

In Ghana, a School Health Education Programme (SHEP) has been in existence for about three decades. The primary aim is to create a well-informed and healthy school population equipped with life skills to maintain healthy habits, attitudes, and behaviors to achieve educational goals (10). The programs are coordinated by teachers selected in each school to serve as SHEP coordinators, while the delivery of services is expected to be provided by the health facilities mapped to each school. At the municipal and educational directorates, officers are assigned to oversee the activities of the various health facilities and SHEP coordinators. Funding for the program primarily comes from the government, and the implementation runs throughout the year while schools are in session. Despite the program’s long-standing operation, there is no published data capturing the coverage and major aspects of health included in the program in various school settings in the country. Furthermore, the inclusion of general and specific eye health interventions in this program remains uncertain. An evaluation of the availability of these interventions in specific community contexts is essential to aid stakeholders in making informed decisions regarding their implementation, sustainability, and effective delivery of the programs. This study was therefore conducted to ascertain the availability of school eye health interventions within the existing School Health Education Program (SHEP) in a Ghanaian municipality.

## Methodology

The study was descriptive cross-sectional and employed a mixed-methods approach. Quantitative and qualitative approaches relevant to addressing the research questions were employed. The total population of the municipality is 93,608 with agriculture being their major occupation. There are 83 public schools in the municipality. The study population included all the 83 basic schools in the municipality, key public health officers and community health nurses engaged in school health in health facilities in the municipality, Municipal School Health Education Programme coordinators at the education and health directorate as well as School-based Health Coordinators. While all the headmasters in all schools available at the time of the study were engaged in the quantitative aspect of the study which focuses on availability, only key informants comprising school health coordinators in the Ghana Education Service and school health workers in the health sector were involved in the in qualitative aspect of the study. All respondents in the study were individuals who were directly involved in the implementation of school eye health interventions. Semi-structured self-administered questionnaires and interview guides (see supplemental material) were used to collect data from headmasters. This involved face to face and telephone call interviews all conducted by the principal investigator. For the Ghana Education Service Officials, Public Health Officials at health facilities and the District Public Health official, data was collected through face-to-face interviews conducted by the principal investigator. Audio recorders where used in cases where the respondent consented. Data was also obtained via an electronic Google form in order to reach those who were not at post at the time of the study. Completeness of data was ensured by marking all essential fields as required. Response summary review, empty cell identification and visual completeness checks were done to ensure data completeness and accuracy. Data was stored on a laptop computer and password protected. All data collected were entered into a spreadsheet and imported into Stata Statistical Software, Version 14.2 for analysis. Categorical data was summarized with frequencies and percentages. Continuous variables were summarized with means and standard deviation. Thematic analysis was done for all qualitative data (Table 1).

**Table 1:** Background of study participants (head teachers).

Variable	Frequency (*N* = 60)	Percentage (%)
Sex		
Male	43	71.67
Female	16	26.67
Undeclared	1	1.67
School type		
Basic school^*^	30	50.00
Junior high school	8	13.33
Primary school	22	36.67
School ownership type		
Private	5	8.33
Public	55	91.67
Community type		
Rural	29	48.33
Semi-urban	17	28.33
Urban	12	20.00
Unspecified	2	3.33

*Basic schools represents schools that are made up of both primary and Junior High but are under the administration of just one management team.

## Results

### Background of study participants

A total of 60 (response rate of 72.3%) heads of schools comprising 43 males and 16 females and 1 undeclared gender took part in the study. This represented the heads of 22 primary schools, 8 junior high schools and 30 basic schools (schools made up of both primary and junior high and with one head). 48% of the schools were located in rural communities while 28 and 20% were located in semi-urban and urban communities, respectively. In terms of school ownership, 92% of the schools were public schools and the rest were privately owned. In addition to the head teachers, 17 school health coordinators and 7 key school health workers were included in the study.

### Availability of school eye health interventions

Of the 60 head teachers who responded, none had a comprehensive eye health program integrated into their school health activities. While 28% of the schools had conducted eye screening programs at some point, these were not part of their regular school health initiatives. Additionally, 25% had provided some form of eye health education, and 32% had ongoing vitamin A supplementation programs. Only 13% of the schools had ever conducted preschool or pre-entrance vision screening as reported by the head teachers (Table 2). Overall, 67% (40 out of 60 schools) had implemented at least one component of a comprehensive eye health program. However, these interventions were sporadic and dependent on external donors or institutions, which implemented them based on the availability of funds.

**Table 2:** Eye health interventions in schools in the Tano North Municipality (as reported by the head teachers).

School health intervention	Basic	Primary	JHS	Total	Percentage
Eye health education					
Available	8	4	3	15	25
Unavailable	22	18	5	45	75
Eye screening					
Available	8	8	1	17	28
Unavailable	22	14	7	43	72
Vitamin A supplementation					
Available	10	8	0	18	30
Unavailable	20	14	8	42	70
Preschool/Pre-entrance vision screening^*^					
Available	6	2	0	8	13
Unavailable	24	20	8	52	87

*Preschool vision screening as used in the study involves screening of children before they are admitted into any school for the first time. Pre-entrance vision screening involves the screening of students when they move from one school to join another school at any level.

In addition to the data from the heads of schools, information was gathered from 17 school health workers (SHWs), consisting of 14 females and 3 males, with an average of 4.5 years of experience (SD: 2.16 years). All SHWs reported that they were not mandated to carry out any eye health interventions in schools within the municipality. Nonetheless, half of them indicated that at least one component of a comprehensive eye health program had been conducted in their respective schools. These interventions, however, were infrequent and irregular, often occurring only once, and were organized by external institutions or organizations. Notably, the SHWs reported that preschool or pre-entrance vision screening was entirely absent in all schools. Key health workers responsible for school health also confirmed the lack of a comprehensive school eye health program as part of their regular activities. Their primary responsibility related to eye care, as reported by them, was vitamin A supplementation, which had been integrated into child welfare clinics held on school premises. Some health workers also reported conducting visual acuity screenings, but this was not a regular practice. Below are some responses from key health workers when asked about eye health interventions in their school health programs:

When asked about their eye health activities, one health worker noted:

*“There is, but it’s just eye screening using the Snellen acuity chart. When we detect abnormalities, we refer them to health facilities for further investigations. We do not provide treatment or any other follow-up. We also do vitamin A supplementation.”* (Key Health Worker 1)

Another two health workers explained their routine

*“We make sure to visit one school each month. During these visits, we do eye screenings, not full examinations. We use the Snellen chart to check if the children can identify the letters. We also provide vitamin A supplementation.”* (Key Health Worker 2)

*“Vitamin A supplementation is mandatory for us. When school resumes, we ensure it is done. Before the program starts, we conduct health education at the community center to explain the importance of the program. We ask parents to bring their children’s health record books. Once at the schools, we register the children and give them vitamin A capsules.”* (Key Health Worker 3)

Vitamin A supplementation, in particular, was well-integrated into the child welfare clinics held on school premises monthly. These clinics, typically run by community health nurses, provided an opportunity to carry out school health programs, with vitamin A supplementation being the most consistently implemented intervention. Before the start of the school year, letters were sent to school heads informing them of the scheduled visits by health workers. In some cases, visual acuity testing was included in the activities, though it was not conducted as frequently or consistently as vitamin A supplementation.

### Human resources used in implementing eye health interventions in schools

**Figure 1:**
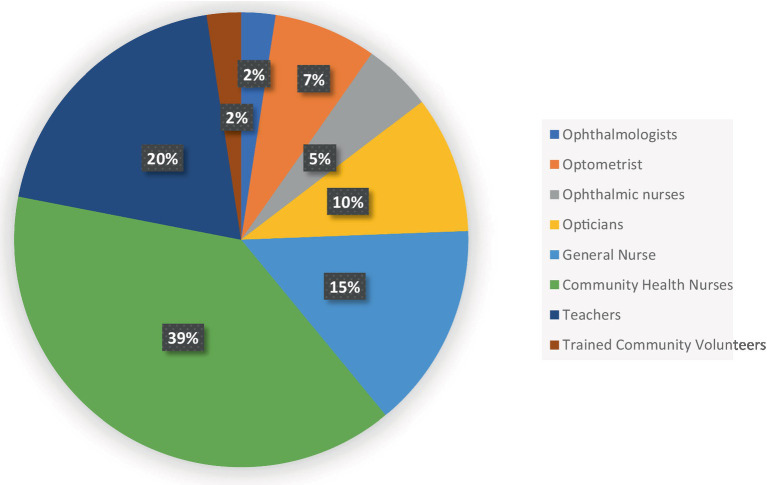
Human resources used in implementing eye health interventions in schools.

Community Health Nurses (CHNs) and teachers were the primary human resources utilized in implementing the available eye health interventions in the municipality. In contrast, optometrists, ophthalmic nurses, ophthalmologists, and trained volunteers were the least involved (Figure 1). The selection of personnel for these programs largely depended on the institutions responsible for or funding the interventions, as well as the specific nature of the programs.

### Sources of funds used for eye health interventions

**Figure 2:**
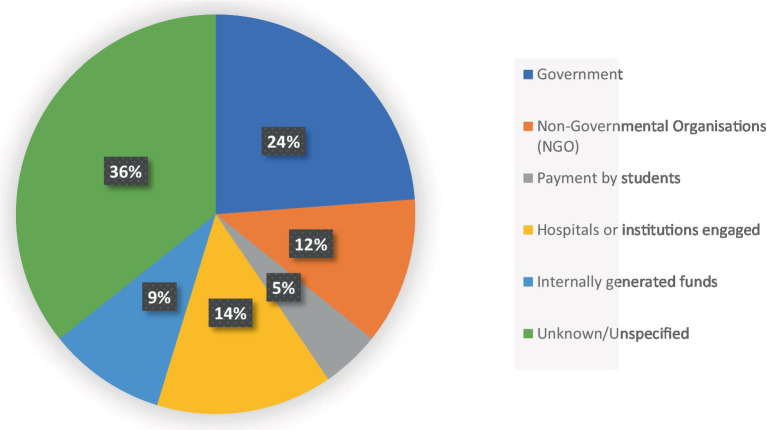
Pie chart showing the sources of funds used for eye health interventions in schools.

More than a third of the respondents, particularly the heads of schools, reported being unaware of the exact sources of funding for the programs implemented in their institutions. Among those who did identify funding sources, the government and the organizations involved in the programs were the primary financiers of the school health interventions (Figure 2). A small number of participants mentioned that individual payments by students and internally generated funds also contributed to the financing of school eye health interventions.

### Protocols for implementation of eye health interventions

**Figure 3:**
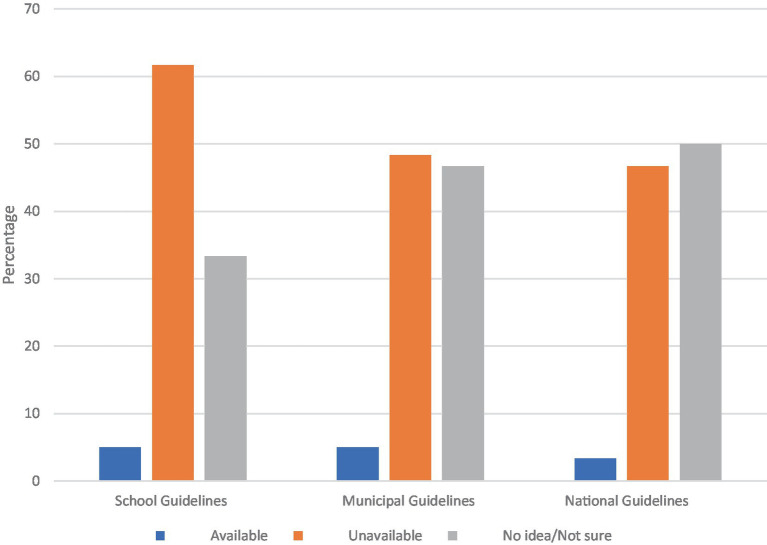
Bar graph showing the availability of guidelines on school eye health interventions as reported by school heads.

All the School Health Education Program (SHEP) Coordinators indicated that they were unaware of any guidelines regarding school eye health interventions at the school, municipal, or national levels. Similarly, 95 % (95%) of the school heads reported that no guidelines were available at any level or that they had no knowledge of their existence. The 5% who stated the guidelines were available were all unable to provide any proof or reference to such a document. As a result, programs were implemented at the discretion of those involved in their execution (Figure 3).

Regarding the procedures for students suspected of having eye conditions, all SHEP Coordinators stated that they typically advise parents to take their children to hospitals for further assessment. While a few respondents reported making efforts to ensure that these students visit health facilities, there were no established systems for following up on referrals. In addition, reports of assessments and interventions are rarely communicated to the relevant teachers and school authorities. Only 25% of the school heads who had conducted at least one health intervention reported receiving a report after the program. Furthermore, only 10% of headmasters indicated that there was a system in place in their schools to ensure that students suspected of having eye conditions visit health facilities, but they were unable to specify what those systems entailed.

### Challenges associated with eye health interventions

Several challenges were identified as barriers to the implementation of school health interventions in the municipality. A significant challenge, as highlighted by one official, is the inadequate allocation of funds:

*“The major challenge that we have with regards to school eye health programs is lack of finances. Close to no allocation of funds is made for the program. It is only sometimes that as part of some projects those funds come for the project period. If funds are there, all the interventions can be implemented regularly.”* (SHEP Coordinator 1)

Additionally, the lack of cooperation from parents was another commonly reported issue, as noted by almost all health officials. This lack of support affects both the implementation of eye health interventions and the follow-up on referrals. Many parents either refuse to allow their children to participate in these interventions due to misconceptions or fail to follow through on referrals to health facilities. One coordinator provided insight into this challenge:

*“One of the major problems we face has to do with some of the kids, I mean those that are referred… (pause)…you know this kind of locality, financial challenges are quite a problem here. So some of them, you speak to them and they tell you ‘my father said he has no money,’ so they did not even go for treatment.”* (SHEP Coordinator 2)

Another official echoed these concerns, emphasizing the specific barriers faced by parents in *following through with referrals:*

*“…even if screenings are done and children are referred, their parents are always reluctant to send them to the health facilities. Firstly, because they complain they will be made to pay out of pocket even if they have NHIS. Secondly, they complain of inability to get money to transport their children to the health facilities.”* (SHEP Coordinator 3)

This sentiment was further reinforced by another health worker, who explained the reluctance of some parents to allow their children to participate in school-based health programs:

“Sometimes when we refer the children, their parents do not send them to the facilities. And some of the times too, the parents do not allow their children to come for the administration to be done. They do not see the need for their wards to receive the capsule, because of their different mind-sets and perceptions.” (Key Health Worker 1)

The study found that no training program has been offered to the teachers and SHEP Coordinators regarding eye health interventions. This coupled with the lack of basic eye testing equipment accounted for the unavailability of some interventions. One SHEP Coordinator described this issue in more detail:

“*For interventions like the preschool and pre-entrance screenings, I think most heads are not aware of it being a major activity to be done when admitting students. Even for those who are aware, there is no training offered to them to allow them conduct such assessments. The knowledge of teachers concerning such interventions may therefore be low. In addition, there are no appropriate materials like the eye testing chart to be used for such assessments.”* (SHEP Coordinator 4)

The study also identified the absence of comprehensive school health teams as a key limitation. In the municipality, school health programs are largely implemented by community health nurses, with minimal support from school health coordinators. However, the lack of specialized personnel such as ophthalmic, dental, and ear-nose-throat (ENT) nurses restricts the scope and delivery of the programs. As noted by one health worker:

*“We all know that there should be a solid school health team. Where we have the eye nurse, a dental nurse, a community health nurse, an environmental health officer, a SHEP coordinator and a medical doctor. But unfortunately we do not have it like that here. So when we (the community health nurses) go (to the schools), we play the role of all the others.”* (Key Health Worker 2)

Finally, the absence of formal guidelines on eye health interventions was cited as another critical challenge. Without clear guidelines, teachers and school health workers lack the necessary knowledge and structure for the implementation of eye health interventions in schools.

## Discussion

The study assessed the availability and implementation of School Eye Health Programs (SEHPs) in a municipality in Ghana, revealing significant gaps in the provision of comprehensive eye health services for schoolchildren. None of the schools in the municipality had a fully implemented SEHP, though over 60% of schools had conducted at least one component of a school eye health intervention, such as vision screening or Vitamin A supplementation, at some point.

The most common interventions identified were vision screenings and Vitamin A supplementation, which were largely conducted by community and public health nurses. However, these interventions were sporadic, often depending on external funding and donor support, with no established consistency or integration into broader school health programs.

Key barriers to the availability and implementation of SEHPs included a lack of financial resources, insufficient training for teachers and school health coordinators, and a lack of specialized health personnel such as ophthalmic nurses or optometrists. Moreover, there were no available guidelines or protocols to standardize SEHP implementation, nor systems to ensure follow-up care or the provision of corrective lenses for children with refractive errors.

### Availability of school eye health programs

The International Agency for the Prevention of Blindness (IAPB), the leading global alliance for the eye health sector, has recently focused considerable attention on child eye health. One notable initiative is the “Focus on Child Eye Health” campaign, introduced in (7). Several countries have implemented various child eye health interventions, particularly in schools. A publication by Abeydeera described how a similar initiative, the “School Medical Inspection (SMI)” program, is being carried out in Sri Lanka (11). A key component of this program is the periodic eye examination to screen for visual problems among school children. Under the most recent enhancement to this program, aligned with VISION 2020, students diagnosed with significant refractive errors are provided with free spectacles, alleviating the financial burden on families. The success of this program is largely attributed to its integration into existing systems, as a result of effective collaboration between Sri Lanka’s Ministry of Health and Ministry of Education. Similar programs have been implemented in various regions worldwide (9, 12–14). While the core aspect of school eye health programs is vision screening to detect and correct refractive errors promptly, such programs must be developed to provide comprehensive and holistic eye care (12, 15). Research has shown that school-based visual acuity screenings are more effective and cost-efficient in delivering refractive error services to students compared to other primary eye care models (6). This study compared eye care services for children delivered through primary eye care setups versus school screening programs, underscoring the importance of adopting school-based screenings, particularly in resource-limited settings like Ghana.

The fact that eye screening programs are available in just over a quarter of schools in the study area highlights the limited access to eye care for most school children. Those who do have access often face higher costs compared to the potential savings if eye care were delivered through school screening programs. To provide cost-effective eye services to school children, the importance of integrated school screening programs cannot be overstated.

One widely implemented eye health intervention in our study area is vitamin A supplementation. This program is fully integrated into child welfare clinic activities at all health facilities. Wherever child welfare clinics are held, including on school premises, vitamin A supplementation is administered alongside other services. This successful integration can serve as a model for implementing other components of school health interventions.

Eye health education is one key components of any comprehensive school eye health programs and carries great impacts (9, 12, 16). In our study, only a quarter of the participating schools had ever conducted any form of eye health education. Even among these schools, the programs were typically one-time events or not considered a significant part of their overall health initiatives. Most school health coordinators do not include eye health education in their routine activities, which tend to focus primarily on general personal hygiene and the administration of medications.

Similarly, reports from health officials involved in school health programs confirmed these findings, noting the absence of eye health education in most schools. One of the key reasons for this gap is the lack of clear guidelines that address all the essential components of the School Health Education Program (SHEP). Although public schools have designated health coordinators, many do not view eye health as a priority within their responsibilities. As a result, it is often excluded from their regular school health education schedules.

Moreover, some health personnel regard eye health as a specialized area that requires the involvement of trained eye health professionals, which further limits its inclusion in school health programs. This points to two major challenges: inadequate training of school health staff and the absence of clear, detailed manuals to guide their work.

Despite these challenges, the widespread presence of Community Health Nurses (CHNs) and School Health Coordinators (SHCs) provides an opportunity. With appropriate training and supervision, these professionals could play a key role in delivering eye health education in schools across Ghana.

### Human resources used in implementing eye health interventions in schools

While it is well established that visual acuity screening is a key component of all school eye health programs, its quality—particularly in identifying conditions like refractive errors that reduce vision—depends significantly on the quality of the program. This quality is largely influenced by the human resources involved and the availability of appropriate standards and guidelines. Visual acuity testing, when conducted without proper history-taking or additional assessments, can result in missed diagnoses of conditions such as latent hyperopia or allergic conjunctivitis, both of which can negatively affect learning outcomes (17). Therefore, it is essential to use appropriately trained personnel to ensure that screening programs are comprehensive (12).

Eye care professionals would ideally be the most suitable workforce for delivering school eye health programs. However, due to their scarcity in many regions, particularly in rural areas, trained teachers have been successfully utilized in school health programs, yielding positive results (18, 19). A study conducted in Ghana assessing the quality of vision screening by teachers found a specificity of 96% and a sensitivity of 76%. This indicates that with proper training, teachers could be a valuable resource in school screening programs, especially in settings like Ghana where there eye care professionals are scarce in rural areas.

In our study, Community Health Nurses (CHNs) were the primary workforce conducting school screenings. Although teachers, particularly school health coordinators, were often supportive during these screenings, they typically did not take an active role. This was due to the lack of targeted training for teachers, except in a few cases where they were trained for one-time projects in some schools. Providing targeted training for both teachers and CHNs in the municipality would facilitate the proper integration of eye screening programs across all schools.

Studies have shown that while non-eye care professionals can be effectively utilized in resource-limited countries, the accuracy of screenings performed by these personnel is often lower compared to screenings conducted by eye care professionals or those with experience in eye health programs (20, 21). It is therefore recommended that individuals with some background or experience in eye care be used whenever possible (20). In cases where such personnel are unavailable, as in our study area, good results can still be achieved if teachers involved in school screenings receive thorough training from qualified eye care professionals. Additionally, the best outcomes are likely when teachers are closely monitored and linked to eye care professionals (22). This approach can improve the sensitivity and specificity of screening tests and ensure timely follow-up and management of refractive errors and other common eye conditions among school children.

### Challenges for implementation of eye health interventions

The International Agency for the Prevention of Blindness has developed a guideline (23) that can be adopted in implementing school eye health programs, particularly in low- and middle-income countries. The adaptation of such guidelines within a school eye health program would ensure consistency and enhance the quality of program delivery. However, none of the schools in our study had any established guidelines or protocols for implementing school eye health programs. This reflects a broader issue; a general lack of such guidelines across the study area and, more concerningly, nationwide. This absence poses a significant challenge, as it could undermine the quality of these programs, even if efforts are made to ensure they are implemented in all schools. There is a clear need to develop or adopt and adapt existing guidelines to support the implementation of school eye health programs in all areas where they are introduced. The lack of guidelines particularly affects follow-up care, which is a critical component for the successful execution of any health program (16).

Currently, no systems are in place to ensure that students referred from screening programs receive the necessary care. As a result, many children diagnosed with eye conditions are left without appropriate interventions. It is crucial to focus on establishing systems that ensure follow-up care when planning the implementation of school eye health programs. Given the strong collaboration between schools and health facilities in the area, equipping major health facilities with eye care personnel and securing funding for follow-up care would ensure that all children receive the necessary treatments.

Other significant challenges hindering the implementation of School Eye Health Programs (SEHPs) in the study area include the lack of reliable funding sources, inadequate targeted training for teachers, limited cooperation from parents, and the absence of established school health teams.

## Conclusion and recommendation

In conclusion, this study highlights significant gaps in the availability and implementation of School Eye Health Programs (SEHPs) in the municipality, with none of the schools offering a fully integrated program despite some individual interventions like vision screenings and Vitamin A supplementation. These interventions, however, lack consistency, as they are largely dependent on external funding and are not systematically integrated into the school health system. Major barriers, including limited financial resources, inadequate training for teachers and health coordinators, and a shortage of specialized eye health personnel, further hinder SEHP implementation. Additionally, the absence of standardized guidelines and protocols exacerbates the challenges, limiting the quality and sustainability of existing interventions. Given the proven success of comprehensive SEHPs in other countries, it is imperative for Ghana’s education and health sectors, alongside other stakeholders, to prioritize the establishment and funding of a cohesive SEHP framework. By addressing these gaps and fostering collaboration between schools, health facilities, and government agencies, Ghana can improve access to essential eye care services for schoolchildren, enhancing both their academic outcomes and overall wellbeing.

## Data Availability

The raw data supporting the conclusions of this article will be made available by the authors, without undue reservation.
