# Supplementary material for: Availability of eye health interventions in basic schools in a Ghanaian municipality
Source: Front Public Health. 2024 Dec 6;12:1468285. doi: 10.3389/fpubh.2024.1468285 (PMC11659203; doi:10.3389/fpubh.2024.1468285)
Supplement: Supplementary file 1 [file Data_Sheet_1.pdf]

AVAILABILITY AND PERCEIVED EFFECTIVENESS OF EYE HEALTH INTERVENTIONS IN SCHOOLS IN THE TANO NORTH MUNICIPALITY.

Dr. Christopher Senyo Adzaho
School of Public Health
College of Health Sciences
Kwame Nkrumah University of Science and Technology

Survey Questionnaire and consent form.

Dear respondent,
I am an MPH, Health Promotion and Education student at the School of Public Health, Kwame Nkrumah University of Science and Technology. As part of the requirements for the award of this degree, I am conducting a study on "The Availability and Perceived Effectiveness of School Eye Health Interventions in the Tano North Municipality". Details of this study is attached in the copy of participants information leaflet in your possessition.

I kindly seek your assistance and cooperation in completing this questionnaire and also granting an interview session. You are assured that any information provided will be kept highly confidential and cannot be traced to you.

Participation in this study is voluntary. You can decide to withdraw at any point in time.

If you agree to partake in this study kindly provide the needed information below.

……………………………	………………………………..	………………………………………..
Signature	Date	Name

Section A: Background of School and Respondent

Please fill by writing or checking all that apply.

1. Sex (*Mark only one oval)*
   - ○ Male
   - ○ Female
   - ○ Other: ______________________

2. What type of school do you head? (*Check all that apply)*
   - ☐ Primary
   - ☐ Junior High School
   - ☐ Senior High School
   - ☐ Other ______________________

3. What type of community is your school located in? (*Mark only one oval)*
   - ○ Rural
   - ○ Semi-Urban
   - ○ Urban
   - ○ Other: ______________________

4. What is the type of school ownership? (*Mark only one oval)*
   - ○ Private
   - ○ Public
   - ○ Other: ______________________

Section B: Availability of Eye health interventions

This section focuses on the current eye health interventions being implemented in your municipality. Kindly answer the questions with all sincerity.

5. Which of the following eye health interventions are available in your school? (Indicate all that apply)
   - ☐ Eye Health Education
   - ☐ Eye Screening
   - ☐ Vitamin A supplementation
   - ☐ Pre-entrance eye screening (for new students)
   - ☐ Preschool vision screening
   - ☐ Other: ______________________

How often is/are the eye health intervention(s) indicated in 6 to 11 done?

6. Eye Health Education (*Mark only one oval)*
   - ○ Annually
   - ○ Biannually
   - ○ Every 3 years
   - ○ Every 4 years
   - ○ Every 5 years and above.
   - ○ Other: ______________________

7. Eye Screening (*Mark only one oval)*

- Annually
- Biannually
- Every 3 years
- Every 4 years
- Every 5 years and above
- Other: ______________________

8. Vitamin A supplementation *Mark only one oval.*

- Annually
- Biannually
- Every 3 years
- Every 4 years
- Every 5 years & above. ______________________
- Other:

9. Pre-entrance eye screening (for new students) (*Mark only one oval)*

- Annually
- Biannually
- Every 3 years
- Every 4 years
- Every 5 years & above.
- Other: ______________________

10. Preschool vision screening (*Mark only one oval)*

- Annually
- Biannually
- Every 3 years
- Every 4 years
- Every 5 years & above.
- Other: ______________________

11. Others. Please specify: ....

Mark only one oval.

- Annually
- Biannually
- Every 3 years
- Every 4 years
- Every 5 years & above.

______________________ o Other:

Section C: Resource for eye health interventions

This section focuses on the resources available for the implementation of eye health interventions. Please answer each of them accordingly

12. Which of the following category of human resource is/are used in delivering the eye health interventions? (*Check all that apply*)

- ○ Optometrists
- ○ Ophthalmologists
- ○ Ophthalmic nurses
- ○ Opticians
- ○ Community Health Nurses
- ○ Teachers
- ○ Trained community volunteers
- ○ General nurses
- ○ Other: ____________________

13. How are the available school eye health interventions financed? (*Mark all that apply)*

- ☐ Funds from government
- ☐ Funds from Non-Governmental Organizations
- ☐ Self-financing by hospitals or institutions engaged in the screening
- ☐ Individual payment by students
- ☐ Internally generated funds
- ☐ Other: ____________________

Section D: Perceived effectiveness of eye health interventions

This section seeks to investigate how effective the current eye health interventions are.

14. Are there any guidelines used in the implementation of eye health interventions in the school? (*Mark only one oval)*

- ○ Yes
- ○ No
- ○ Not sure

15. If you chose "yes" to the question (14) above, cite a reference to the guidelines used:

____

____

____

____

16. Are there available guidelines for school eye health interventions in your municipality? (*Mark only one oval)*

- ○ Yes
- ○ No
- ○ Not sure

17. If you chose "yes" to the question (16) above, cite a reference to the guidelines used:

____

____

____

____

18. Are there available guidelines for school eye health interventions in your nationally? (*Mark only one oval)*

- ○ Yes
- ○ No
- ○ Not sure

19. If you chose "yes" to the question (18) above, cite a reference to the guidelines used:

____

____

____

____

20. Have you ever had a report of any of the eye health interventions carried out in the school? (*Mark only one oval)*

- ○ Yes
- ○ No

21. If you chose "yes" to question above, what information was included in the report? *(Mark all that apply)*

- ☐ Number of students covered by the intervention
- ☐ Major findings from screening programs
- ☐ Referral information of those that required referrals
- ☐ Detailed information of facilities referrals is made to
- ☐ Recommendations
- ☐ Other: ______________________________

22. Is there a system in place to ensure students who are referred from screening programs visit eye care facilities for management? (*Mark only one oval)*

- ○ Yes
- ○ No
- ○ I have no idea

If yes, please give details: ………………………………………………………………………………..

………

Thank you for participating in this survey
