# Supplementary material for: Availability of eye health interventions in basic schools in a Ghanaian municipality
Source: Front Public Health. 2024 Dec 6;12:1468285. doi: 10.3389/fpubh.2024.1468285 (PMC11659203; doi:10.3389/fpubh.2024.1468285)
Supplement: Supplementary file 2 [file Data_Sheet_2.pdf]

AVAILABILITY AND PERCEIVED EFFECTIVENESS OF EYE HEALTH INTERVENTIONS IN SCHOOLS IN THE TANO NORTH MUNICIPALITY.

Dr. Christopher Senyo Adzaho
School of Public Health
College of Health Sciences
Kwame Nkrumah University of Science and Technology

Survey Questionnaire and consent form.

Dear respondent,
I am an MPH, Health Promotion and Education student at the School of Public Health, Kwame Nkrumah University of Science and Technology. As part of the requirements for the award of this degree, I am conducting a study on "The Availability and Perceived Effectiveness of School Eye Health Interventions in the Tano North Municipality". Details of this study is attached in the copy of participants information leaflet in your possessition.

I kindly seek your assistance and cooperation in completing this questionnaire and also granting an interview session. You are assured that any information provided will be kept highly confidential and cannot be traced to you.

Participation in this study is voluntary. You can decide to withdraw at any point in time.

If you agree to partake in this study kindly provide the needed information below.

………………………………	………………………………..	………………………………………..
Signature	Date	Name

QUESTIONNAIRE: AVAILABILITY AND PERCEIVED EFFECTIVENESS OF EYE HEALTH INTERVENTIONS IN SCHOOLS IN THE TANO NORTH MUNICIPALITY.

BACKGROUND OF RESPONDENT

Please fill by writing or checking all that apply.

1. Sex

   Mark only one oval.

   ◯ Male

   ◯ Female

   ◯ Other: ………………………………………………………………………….

2. What role do you play in the health directorate or in your facility?

   …………………………………………………………………………………………………
   …………………………………………………………………………………………….

3. Does your work involve working with schools in the municipality?

   Mark only one oval.

   ◯ Yes

   ◯ No

4. What specific role are you expected to play with regards to school health interventions in the municipality?

   …………………………………………………………………………………………………
   …………………………………………………………………………………………….............

5. How long have you served in your current position in the municipality? ………………….

Availability of Eye health interventions

This section focuses on the current eye health interventions being implemented in your municipality. Kindly answer the questions with all sincerity.

6. Which of the following eye health interventions are available in schools in the municipality? (Mark all that apply)

- ☐ Eye Health Education (How often?...)
- ☐ Eye Screening How often? (How often..)
- ☐ Vitamin A supplementation (How often?..)
- ☐ Pre-entrance eye screening (for new students) (How often?..)
- ☐ Other:.. (How often ....)

Resource for eye health interventions

This section focuses on the resources available for the implementation of eye health interventions. Please answer each of them accordingly

7. Which of the following category of human resource is/are used in delivering the eye health interventions? (***Mark all that apply***)

- ☐ Optometrists
- ☐ Ophthalmologists
- ☐ Ophthalmic nurses
- ☐ Opticians
- ☐ Community Health Nurses
- ☐ Teachers
- ☐ Trained community volunteers
- ☐ General Nurses
- ☐ Other: ______________________________

8. How are the available school eye health interventions financed?

Check all that apply

- [ ] Funds from government
- [ ] Funds from Non-Governmental Organizations
- [ ] Self- financing by hospitals or institutions engaged in the screening
- [ ] Individual payment by students
- [ ] Internally generated funds
- [ ] District Assembly Common Fund
- [ ] Funds allocated to the Municipal Health Directorate
- [ ] Other (Please Specify): ____________________

Perceived effectiveness of eye health interventions

This section seeks to investigate how effective the current eye health interventions are.

9. Are there any guidelines used in the implementation of eye health interventions in the schools? (***Mark only one oval***)

- ( ) Yes
- ( ) No
- ( ) Not sure

10. If you chose "yes" to the question 14 above, cite a reference to the guidelines used:

____

____

11. Are there available guidelines to be used for school eye health interventions in your municipality? (***Mark only one oval***)

- ( ) Yes
- ( ) No
- ( ) Not sure

12. If you chose "yes" to the question 11 above, cite a reference to the guidelines used:

____

13. Are there available guidelines to be used for school eye health interventions nationally? ***(Mark only one oval)***

- ⬭ Yes
- ⬭ No
- ⬭ Not sure

14. If you chose "yes" to the question 13 above, cite a reference to the guidelines used:

____

____

15. Have you ever had a report of any of the eye health interventions carried out in schools in the municipality? (***Mark only one oval***)

- ⬭ Yes
- ⬭ No

16. What information was included in the report? (***Mark all that apply)***

- ☐ Number of students covered by the intervention
- ☐ Major findings from screening programs
- ☐ Referral information of those that required referrals
- ☐ Detailed information of facilities referrals is made to
- ☐ Recommendations
- ☐ Other: ______________________________

Thank you for participating in this survey
