# Supplementary material for: Availability of eye health interventions in basic schools in a Ghanaian municipality
Source: Front Public Health. 2024 Dec 6;12:1468285. doi: 10.3389/fpubh.2024.1468285 (PMC11659203; doi:10.3389/fpubh.2024.1468285)
Supplement: Supplementary file 3 [file Data_Sheet_3.pdf]

INTERVIEW GUIDE

AVAILABILITY AND PERCEIVED EFFECTIVENESS OF EYE HEALTH INTERVENTIONS IN SCHOOLS IN THE TANO NORTH MUNICIPALITY

Availability and Perceived Effectiveness of Eye Health Interventions

1. What school eye health interventions are schools in the municipality expected to include in school health programs?

2. Are all the interventions currently being implemented?

3. What are the major components in the implementation of intervention that you have mentioned as currently available? What exactly do they entail? What laid down procedures are involved in their implementation?

4. What arrangements are in place for students who are suspected of various health conditions or who fail screening tests?

5. Is there currently a system in place to ensure students who are referred from screening programs visit eye care facilities for management?

   If yes, kindly state clearly the systems that is in place.

6. Is there any system in place where spectacles are provided for students with refractive errors? If yes, kindly state clearly the systems that is in place.

7. How effective do you think the school screening programs and other eye health intervention in the Tano North Municipality are?

8. What is the reason for your answer above?

9. Does the health facilities, health directorate and the education directorate have any collaboration for the implementation of the interventions? Give details.

10. What accounts for the choice of human resource in the implementation?

CHALLENGES AND RECOMMENDATIONS

This section seeks to investigate the current gains, challenges as well as recommendations for implementing or improving the eye health interventions in the municipality

11. Are there any achievements in implementing eye health interventions you would like to talk about?
12. What do you think accounted for the achievements?
13. What are the challenges you have encountered in implementing the eye health interventions?

14. For the interventions like … which you indicated in the questionnaire you just filled as not available currently, what is the reason for their unavailability?

15. What will your recommendations to anyone who will like to roll out eye health intervention programs in schools in your municipality?

Thank you for participating in this survey
